# Cognitive Assessment in SARS-CoV-2 Patients: A Systematic Review

**DOI:** 10.3389/fnagi.2022.909661

**Published:** 2022-07-01

**Authors:** Bruno Biagianti, Asia Di Liberto, Aiello Nicolò Edoardo, Ilaria Lisi, Letizia Nobilia, Giulia Delor de Ferrabonc, Elisa R. Zanier, Nino Stocchetti, Paolo Brambilla

**Affiliations:** ^1^Department of Neurosciences and Mental Health, Fondazione IRCCS Ca’ Granda, Ospedale Maggiore Policlinico, Milan, Italy; ^2^Department of Pathophysiology and Transplantation, University of Milan, Milan, Italy; ^3^University of Milano-Bicocca, Milan, Italy; ^4^School of Medicine and Surgery, University of Milano-Bicocca, Monza, Italy; ^5^Laboratory of Acute Brain Injury and Therapeutic Strategies, Department of Neuroscience, Istituto di Ricerche Farmacologiche Mario Negri IRCCS, Milan, Italy; ^6^Department of Psychology, University of Milano-Bicocca, Milan, Italy; ^7^Department of Anaesthesia and Critical Care, Fondazione IRCCS Ca’ Granda Ospedale Maggiore Policlinico, Milan, Italy

**Keywords:** SARS-CoV-2, COVID-19, neuropsychology, psychometrics, cognitive impairment

## Abstract

**Background:**

Patients with post-infective severe acute respiratory syndrome coronavirus 2 (SARS-CoV-2) often show both short- and long-term cognitive deficits within the dysexecutive/inattentive spectrum. However, little is known about which cognitive alterations are commonly found in patients recovered from SARS-CoV-2, and which psychometric tools clinicians should consider when assessing cognition in this population. The present work reviewed published studies to provide a critical narrative of neuropsychological (NPs) deficits commonly observed after SARS-CoV-2 infection and the tests most suited for detecting such cognitive sequelae depending on illness severity.

**Methods:**

This review followed the Preferred Reporting Items for Systematic reviews and Meta-Analyses (PRISMA) guidelines and was pre-registered on Prospective Register of Systematic Reviews (PROSPERO) (CRD42021253079). Observational studies quantitatively assessing cognition in patients with post-infective SARS-CoV-2 were considered. From 711 retrieved articles, 19 studies conducted on patients with SARS-CoV-2 without medical comorbidities were included and stratified by disease severity.

**Results:**

The majority of studies (*N* = 13) adopted first-level tests. The most frequently administered screeners were the Montreal Cognitive Assessment (MoCA) and the Mini-Mental State Examination (MMSE)—with the former more likely to detect mild, and the latter moderate/severe deficits. Among second-level tests, those assessing attention and executive functions (EFs) were highly represented. Remotely-delivered tests yielded lower percentages of cognitive impairment. Overall, cognitive domains often found to be impaired were EFs, attention, and memory.

**Conclusion:**

Cognitive sequelae in patients with post-infective SARS-CoV-2 can be detected with NPs testing. Depending on the psychometric test features, the likelihood of observing cognitive deficits can vary. Further studies on larger sample sizes are needed to investigate the clinical usefulness of second-level tools. The primary goal of preventative health services should be the early detection and intervention of emerging cognitive deficits.

## Key Points

- Cognitive sequelae are prevalent in patients with SARS-CoV-2, while the likelihood of observing such sequelae varies depending on the test used.
- Among patients with SARS-CoV-2, MoCA is more likely to detect mild cognitive deficits, whereas MMSE moderate/severe deficits.
- Studies using domain-specific tests are needed, to investigate whether some specific cognitive functions are more impaired than others.
- A standardized protocol for cognitive assessment in patients with SARS-CoV-2 should be made available to clinicians.

## Introduction

The novel human-infecting coronavirus (severe acute respiratory syndrome coronavirus 2 [SARS-CoV-2]) causes a multi-organ disease (COVID-19) that can impact the central nervous system (CNS; Coolen et al., 2020; Boscutti et al., 2021). Coronaviruses are known to elude the immune response and spread to cells other than those of the respiratory tract and have shown the ability to be neuro-invasive (Xu et al., 2005; Arabi et al., 2015). Several mechanisms by which SARS-CoV-2 can damage the CNS have been hypothesized. These include direct infection, viruses entering through blood circulation and neuronal pathways, hypoxic and immune injury, as well as binding to the angiotensin-converting enzyme 2 (ACE2) receptor (Baig et al., 2020). The neurotropism of SARS-CoV-2 allows it to escape the host immune response and achieve latency, which possibly causes both acute and long-term neurological effects, such as cognitive dysfunction (Blomberg et al., 2021). Indeed, post-mortem studies have found brain alterations among patients deceased because of COVID-19. Specifically, subcortical microbleeds and macrobleeds, asymmetric olfactory bulbs, and ischemic lesions have been observed through structural brain magnetic resonance imaging (Coolen et al., 2020). Furthermore, post-mortem histological/immunohistochemical analyses revealed the presence of astrogliosis in several regions (e.g., olfactory bulb, basal ganglia, and cerebellum), activation of microglia, and infiltration of cytotoxic T lymphocytes primarily in the cerebellum and brainstem (Matschke et al., 2020). Nonetheless, our understanding of such mechanisms remains limited, and most of the available evidence comes from previous SARS-CoV infections, post-mortem studies, and mouse transgenic models (Bao et al., 2020).

Health clinics are seeing an influx of patients with cognitive problems who were otherwise healthy prior to COVID-19 infection (Esposito et al., 2021; Nersesjan et al., 2022). From the emerging evidence and current understanding of the mechanism of SARS-CoV-2 action in the CNS, one can expect to a range of cognitive impairments that can either occur during the acute phase or manifest as long-term sequelae. Regarding short-term complications, deficits in working memory (WM), set-shifting, divided attention, and processing speed have been reported, with most patients showing mild-to-moderate symptoms (Varatharaj et al., 2020). Presently, we have limited ability to discuss the long-term cognitive consequences of COVID-19. However, in line with structural brain alterations found post-mortem across deceased patients, along with neuroimaging alterations found in COVID-19 patients with cognitive deficits (Douaud et al., 2022), we can expect that COVID-19 survivors would show long-term cognitive difficulties. Therefore, the cognitive evaluation of patients with COVID-19 should include first-level tests—i.e., screeners that usually provide a global index of general cognitive functioning—as well as second-level tests—i.e., tests that are able to provide an accurate evaluation of domain-specific cognitive functions, such as attention, speed of processing, executive functions (EFs), learning, and memory.

Given the past outbreaks of coronaviruses as well as current reports of COVID-19-related neurological complications, a large number of patients with COVID-19 will likely experience cognitive symptoms during or after the active phase, which will in turn negatively affect their psycho-social and functional outcomes (Jacobs et al., 2020). For these reasons, several studies have attempted to identify and characterize early cognitive sequelae associated with COVID-19 (Douaud et al., 2022). A detailed and longitudinal evaluation should be always considered in COVID-19 patients with cognitive complaints to monitor the emergency, the frequency, the severity, and subject-specific profile of cognitive dysfunction, given the high rate of inter-individual variability. This heterogeneity is primarily due to contextual factors that are known to impact cognition. First, the severity of SARS-CoV-2 infection, along with its medical management, seems to affect cognitive outcomes. As a matter of fact, a higher rate of cognitive impairment was found among patients with COVID-19 who experienced delirium relative to those without delirium (Mcloughlin et al., 2020). Second, hypoxemic respiratory failure, duration of intubation, or time elapsed from extubation to assessment are all known to impact cognitive performance (Turon et al., 2018; Sasannejad et al., 2019)—although a recent study did not find significant associations between the type of ventilation and cognitive impairment (Jaywant et al., 2021). Additionally, while the premorbid cognitive status of individuals who recovered from COVID-19 is often unknown, possible pre-existing cognitive dysfunction, age, and general medical comorbidities impairing cognition may all play a pivotal role (Gunstad et al., 2010; Wu et al., 2011; Seliger et al., 2015; Kim et al., 2016). As a matter of fact, lower cognitive ability was found to be a key risk factor associated with the likelihood of SARS-CoV-2 infection/hospitalization (Batty et al., 2020).

Other aspects that are likely responsible for the high degree of heterogeneity in cognitive dysfunction include elements associated with cognitive evaluation: first-level and second-level tests may have different psychometric and diagnostic properties toward COVID-19-related cognitive impairment (Block et al., 2017), similar to how remote and in-person administration might not always elicit comparable results (Bilder et al., 2020).

The purpose of this systematic review is to identify which NPs (NPs) tests are best able to capture the cognitive complications following COVID-19. First, we review all published articles that included all first- and second-level NPs testing. Second, we classify these findings based on disease severity, so that it becomes possible to determine which test is most useful to characterize a specific cognitive domain at a given level of illness severity. Third, for each test, we report the percentage of patients with deficits. Finally, we note differences between in-person *vs.* remote administration, when available.

## Methods

The present systematic review was performed according to the Preferred Reporting Items for Systematic reviews and Meta-Analyses guidelines (PRISMA, Page et al., 2021); PRISMA checklist is provided in Supplementary Table 1.

This systematic review was pre-registered on the International Prospective Register of Systematic Reviews (PROSPERO)—identification number: CRD42021253079 (https://www.crd.york.ac.uk/PROSPERO/display_record.php?RecordID=253079).

### Search Strategy

The online search strategy was conducted on 30 October 2021 through two of the major public scientific databases, PubMed and Scopus. The following search terms were entered: (“COVID-19” OR “SARS-CoV-2” OR “coronavirus”) AND (“cognitive impairment” OR “cognitive deficit” OR “neuropsychology”). For Scopus, the fields of search were title, abstract, and keywords; for PubMed, the fields of search were title and abstract only. Additional studies that were manually retrieved have been included. No date limit was set and only contributions written in English were included. Gray literature was not searched for.

### Inclusion and Exclusion Criteria

Observational studies (cross-sectional and longitudinal) quantitatively assessing patients with COVID-19 for different modalities, components, and functions of cognition by means of standardized tests were considered for eligibility. Abstracts, reviews, meta-analyses, opinion papers, research protocols, qualitative studies, case series studies, articles with no standardized tests administered to patients with COVID-19, and articles that present samples with severe comorbidities known to impact cognitive functioning were excluded.

### Bias Assessment

Formal quality assessment was performed by four independent raters (AD, IL, LN, and GF) by means of the Standard Quality Assessment Criteria (SQAC, Kmet et al., 2004). Disagreements were solved *via* discussion with a fifth independent rater (BB). Non-applicable items were removed from the SQAC (range = 0–20).

### Study Selection Process and Data Collection

**FIGURE 1:**
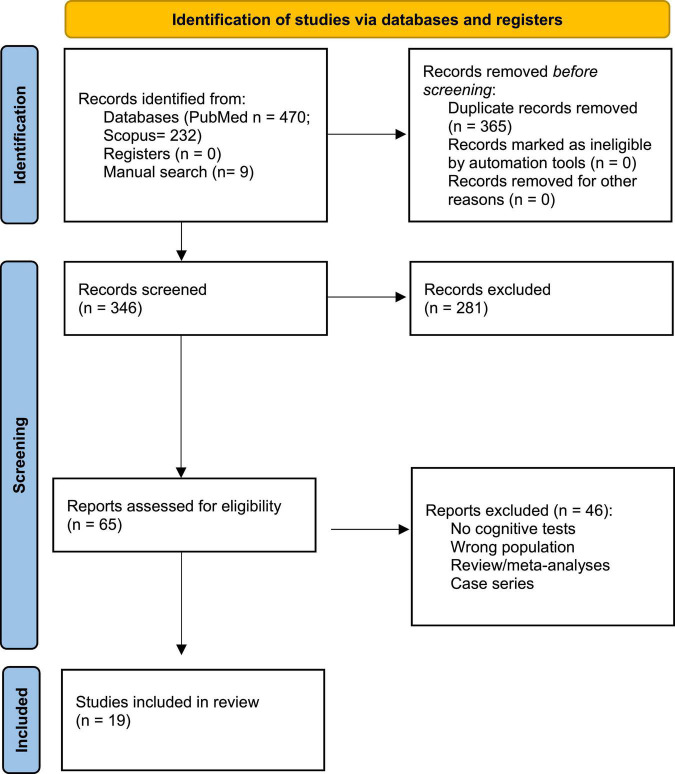
PRISMA 2020 flow diagram for the systematic review, which included searches of Pubmed and Scopus databases.

The study selection process is shown in Figure 1.

The search, conducted from May 2021 to October 2021, provided 711 potentially relevant articles. After the removal of duplicates, 346 articles were available for screening—along with nine articles identified through manual search. The screening was performed independently by three of the authors (AD, IL, and LN) who were blinded to each other’s decisions *via* Rayyan^1^. Disagreements were resolved by reaching a consensus. From the initial pool, 65 articles were then assessed for eligibility, of which 46 were excluded based on exclusion criteria. A total of 19 studies were included in this review. Taken together, the studies included in this review assessed 1.197 patients infected by SARS-CoV-2.

Data extraction was performed by four independent Authors (AD, IL, LN, and GF), whereas a fifth independent rated (BB) checked the extracted data and resolved disagreements. The following variables were extracted from included studies: authors and year; study design (cross-sectional *vs.* longitudinal); number of patients; age; education; sex; disease severity and duration; time between infection and assessment; modality of assessment (in person *vs.* remote); tests that were administered; first- *vs.* second-level assessment; cognitive domains or behavioral aspects that were assessed; and scores on NPs tests.

## Results

### Study Categorization

In light of the high heterogeneity in COVID-19 severity, included articles were stratified according to disease severity to better understand the prevalence and nature of cognitive deficits. Studies were stratified as follows: severe, if patients required intensive care unit (ICU) admission and/or invasive ventilation (*N* = 5); moderate, if patients required hospitalization (*N* = 3); and mild, if no hospitalization was needed (*N* = 1). Whenever a study included patients with different degrees of severity, or severity was not specified, the study was categorized as having a mixed population (*N* = 10).

### Outcome Overview

A summary of the included articles and their data are provided in Table 1. Five studies investigated severe patients, three moderate patients, and one mild patients, whereas 10 featured patients with mixed or unspecified severity.

**TABLE 1:** List of included studies.

References	Study type	*N* (diagnosis modality)	Age (year)	Sex% males	Education (years)	Disease severity	Disease duration	Time of assessment from onset	Assessment modality	Assessment level	Cognitive test: % of patients with deficit (n of patients with deficit/N)
Aiello et al., 2022	Cross sectional	100 of which 55 RCD + and 45 RCD- (not reported)	*RCD*+: 66.13 ± 13.84 *RCD–:* 63.33 ± 11.4	*RCD*+: 61% M *RCD–:* 86% M	*RCD*+: 11.2 ± 3.63 *RCD–:* 11.02 ± 3.89	Mixed	40.6 ± 26.72 (2–113) [days] 42.31 ± 26.26 (5–129) [days]	74.13 ± 41.02 (7–241) [days] 76.43 ± 35.33 (26–186) [days]	In person	I level	*RCD*+: MMSE: 20% (11/55) MOCA: 23.6% (13/55) *RCD–:* MMSE: 2.2% (1/45) MOCA: 4.4% (2/45)
Alemanno et al., 2021	Longitudinal	87 (PCR)	67.23 ± 12.89	71% M	N/A	*N* = 31 severe *N* = 47 moderate (18 BPAP, 29 Venturi Mask) *N* = 9 mild	12.39 ± 6.51 [intubation; days] N/A N/A N/A	5–20 days	In person	I level	*Severe:* MMSE: 12.9% (4/31) MoCA: 72% (22/31) *Moderate (BPAP):* MMSE: 55.6% (10/18) MoCA: 94.4% (17/18) *Moderate (Venturi Mask):* MMSE: 48.3% (14/29) MoCA: 89.6% (26/29) *Mild*: MMSE: 44.4% (4/9) MoCA: 77.8% (7/9)
Almeria et al., 2020	Cross-sectional	35 (PCR)	47.6 ± 8.9	45.7% M	12.6 ± 4.6	Mixed	10.8 ± 9.2 [days]	10–35 days after hospital discharge	In person	II level	TAVEC: 2.9% (1/35) WMS-IV: no deficit Digit Forward: no deficit Digit Backwards: 8.6% (3/35) letter and numbers: no deficit TMT A: 2.9% (1/35) TMT B: 8.6% (3/35) SDMT: 5.7% (2/35) Stroop: 2.9% (1/35) Phonemic Fluency: 11.4% (14/35) Semantic Fluency: 5.7% (2/35) BNT: 2.9% (1/35)
Boesl et al., 2021	Cohort	100 (PCR or antibodies)	45 [20–79]	33% M	N/A	Mixed	N/A	184.5 [days]	In person	I level	MoCA: 30% (30/100)
Del Brutto et al., 2021	Longitudinal	52 (serology)	59.4 ± 10.6	38% M	N/A	Mild	N/A	N/A	In person	I level	MoCA: 21% (11/52)
Ferrucci et al., 2021	Cross-sectional	38 (not reported)	53.45 ± 12.64	71% M	12.39 ± 3.24	Moderate	9.84 ± 3.95 [days of hospitalization]	132.86 ± *36.62*	In person	II level	BRB-NT: 60.5% (23/38) SRT: 26.3% (10/38) SPART: 15.8% (6/38) SDMT: 42.1% (16/38) PASAT: 10.5% (4/38) WLG: 7.9% (3/38)
Heyns et al., 2021	Cross-sectional	135 of which 38 assessed cognitively (PCR)	72.0 [58.0–86.0]	49.6% M	N/A	*N* = 15 severe *N* = 23 moderate	>7 days of hospitalization	N/A	In person	I level	*Severe:* MoCA: 46.7% (7/15) *Moderate:* MoCA: 60.9% (14/23)
Lamontagne et al., 2021	cohort	100 of which 50 COVID-19 patients (PCR) and 50 HCs	*COVID*: 30.8 ± 7.79 *HC:* 29.14 ± 9.87	*COVID*: 42% M *HC:* 28% M	*COVID*: N/A *HC:* N/A	mixed	N/A	123.63 ± 94.71 [days post diagnosis]	Remote	II level	ANT:% N/A
Latronico et al., 2022	Longitudinal	114 (admission to ICU)	60 [52-66]	77% M	N/A	Severe	N/A	3, 6, and 12 months post discharge	In person	I level	*3 months post discharge* MoCA: 25% (25/98) *6 months post discharge* MoCA: 22% (17/77) *12 months post-discharge* MoCA: 13% (7/51)
Mazza et al., 2021	Longitudinal	130 cognitively assessed (PCR)	58.85 ± 12.8	66% M	12.58 ± 3.68	Mixed	N/A	90.1 ± 13.4 [days] after hospital discharge	In person	II level	BACS: 16% (21/130) deficit in at least one function 17% (22/130) deficit in at least 2 functions 14% (18/130) deficit in at least 3 functions 10% (14/130) deficit in at least 4 functions 5% (7/130) deficit in at least 5 functions Executive functions: 50% of impaired patients Psychomotor coordination: 57% of impaired patients
Mcloughlin et al., 2020	Longitudinal	71 (PCR)	61 [24–91]	72% M	N/A	Mixed	N/A	N/A	Remote	I level	TICS-m:% N/A
Miskowiak et al., 2021	Longitudinal	29 (PCR)	56.2 ± 10.6	59% M	14.3 ± 3.9	Moderate	N/A	3 months post hospital discharge	In person	II level	SCIP-D: 59–65% VLT-L:% N/A WMT:% N/A VFT:% N/A VLT-D:% N/A PMT:% N/A TMT-B:% N/A
Monti et al., 2021	Longitudinal	39 (PCR)	56 ± 10.5	89% M	N/A	Severe	23–44 days in hospital 7–16 days in ICU	51–71 days after ICU discharge	Remote	I level	Itel-MMSE: 2.6% (1/39)
Patel et al., 2021	Cohort	77 (not reported)	61.3 ± 15.67	36.4% M	N/A	Mixed	37.03 ± 31.8 [days]	N/A	In person	I level	MoCA: 80.5% (62/77)
Pilotto et al., 2021	longitudinal	126 (hospitalization)	64.8 ± 12.6	50% M	N/A	Mixed	11.6 ± 8.8	6 months after hospital discharge	In person	I level	MoCA: 17.5% (22/126)
Pistarini et al., 2021	Cross sectional	27 of which 20 COVID-19 positive and 7 post-COVID patients (nasal swab)	64.13 ± 15.85	37.5% M	11.15 ± 4.88	Severe	N/A	10 days after symptom onset N/A	In person	I level	*COVID-19 positive:* MMSE: 35% (7/20) *Post-COVID:* MMSE: 5% (1/7)
Solaro et al., 2021	Cohort	32 (nasal swab)	53.77 ± 4.81	59% M	N/A	moderate	16.54 ± 9.08 [days]	N/A	In person	I level	MoCA: 36.7% (13/32) Mean MoCA score: 20(8)
Soldati et al., 2021	Cross-sectional	23 (not reported)	53.6 ± 11.7	78% M	12.7 ± 3.5	Severe	12.3 ± 7 [days; ICU stay]	37–115 [days]	Remote	I level	TICS: 13% (3/23)
Zhou et al., 2020	Cross-sectional	29 (recovered from COVID-19)	47 ± 10.54	62% M	12.59 ± 2.78	Severe	N/A	N/A	Remote	II level	TMT: no deficit SCT: no deficit CPT:% N/A DST: no deficit

*M, male; PCR, Polymerase Chain Reaction; RCD+, at risk for cognitive deficits; RCD–, not at risk for cognitive deficits; MMSE, Mini Mental State Examination; MoCA, Montreal Cognitive Assessment; FAB, Frontal Assessment Battery; N/A, not available; itel-MMSE, Italian telephone version of MMSE; TICS, telephone interview for cognitive status; BPAP, Biphasic Positive Airway Pressure; SCIP-D, Screen for Cognitive Impairment in Psychiatry Danish Version; VTL-L: VLT-L, verbal learning test-learning; WMT, working memory test; VFT, verbal fluency test; VLT-D, verbal learning test-delayed recall; PMT, psychomotor speed test; TMT-B, Trail Making Test B; BRB-NT, Brief Repeatable Battery of Neuropsychological Tests; SRT, Selective Reminding Test; SPART, Spatial Recall Test; SDMT, Symbol Digit Modalities Test; PASAT, Paced Auditory Serial Addition Test; WLG, Word List Generation Test (WLG); TAVEC, Test de Aprendizaje Verbal Espa na-Complutense; WMS-IV, Wechsler Memory Scale –IV; BNT, Boston Naming Test; BACS, Brief Assessment of Cognition in Schizophrenia; SCT, Sign Coding Test; CPT, Continuous Performance Test; DST, Digital Span Test; TICS-m, Modified Telephone Interview for Cognitive Status.*

The mean SQAC scores was 17.8/20 ± 1.8/20 (17/18 for articles with non-applicable items). In 14 studies, NPs’ assessment took place in person, while five studies tested patients remotely. In one study the assessment took place both in-person and remotely.

In total, 13 studies used a first-level assessment tool, with the Montreal Cognitive Assessment (MoCA; Nasreddine et al., 2005) being the most frequently administered test, followed by the Mini Mental State Examination (MMSE; Folstein et al., 1975), and the Telephone Interview for Cognitive Status (TICS; Brandt et al., 1988). Additionally, six studies investigated specific NPs domains with second-level assessments.

### Overview of Neuropsychological Assessment in Patients With COVID-19

#### Neuropsychological Assessment in Severe Patients

Within articles including *severe* patients (*N* = 5), two administered the NPs evaluation in person while three did it remotely.

Furthermore, four out of five studies used a first-level assessment, with MoCA and MMSE being the most commonly used, followed by TICS. One study (Zhou et al., 2020) used the following second-level tests: Trail Making Test (TMT), Sign Coding Test (SCT), Continuous Performance Test (CPT), and Digit Span Test.

In addition, two studies from mixed categories reported NPs scores separately for severe patients (Alemanno et al., 2021; Heyns et al., 2021): their findings are therefore reported in this section. Both studies assessed cognition through in-person evaluation, administering MoCA (Alemanno et al., 2021; Heyns et al., 2021), and MMSE (Alemanno et al., 2021), respectively.

All studies investigating global cognition in severe patients with the MoCA encompassed in-person assessments and identified pathological scores in 46% (7 out of 15 patients, Heyns et al., 2021) and 70% (22 out of 31 patients, Alemanno et al., 2021) of patients, respectively. Latronico et al. (2022) assessed patients longitudinally after hospital discharge and found that 25% (25/98) of patients were pathological on MoCA at 3 months after discharge, 22% (17/77) pathological after 6 months, and 13% (7/51) pathological after 12 months.

The MMSE was administered both in-person (Alemanno et al., 2021; Pistarini et al., 2021) and remotely (Monti et al., 2021). Scores on MMSE highlighted relatively low yet the variable prevalence of pathological scores—specifically 13% (4 out of 31, Alemanno et al., 2021) and 2.5% (1 out of 39, Monti et al., 2021). Pistarini et al. (2021) divided their sample into patients with acute COVID-19 and post-COVID, and found cognitive deficits in 35% (7 out of 20) and 5% (1 out of 7), respectively. It is worth noting that Alemanno et al. (2021) administered both MoCA and MMSE to the same patients, revealing different proportions of impairment when using the two tests.

The study, such as the TICS reported that only 3 out of 23 patients (13%) had pathological scores (Soldati et al., 2021).

Finally, in Zhou et al. (2020), patients with COVID-19 showed cognitive deficits in sustained attention, assessed with the CPT. When compared with healthy controls, patients with COVID-19 showed lower correct number and higher missing numbers on CPT 2 and CPT 3, error detection rate, and missed detection rate.

#### Neuropsychological Assessment in Moderate Patients

Studies with samples of *moderate* severity (*N* = 3) all performed in-person NPs assessments; one study used the first-level (MoCA, Solaro et al., 2021) and two studies used the second-level (Ferrucci et al., 2021; Miskowiak et al., 2021) tests. The two studies from mixed categories reported NPs outcomes separately for moderate samples (Alemanno et al., 2021; Heyns et al., 2021): their findings are therefore reported in this section. Both studies assessed cognition through in-person evaluation administering MoCA (Alemanno et al., 2021; Heyns et al., 2021) and MMSE (Alemanno et al., 2021).

Studies administering the MoCA test found pathological scores in 60% (14 out of 23, Heyns et al., 2021) and 36% (13 out of 32; Solaro et al., 2021) of patients. Alemanno et al. (2021) further subdivided moderate patients into those requiring Bilevel Positive Airways Pressure (BPAP) ventilation or Venturi mask; they found MoCA deficits in 94% of those with BPAP (17 out of 18), and in 89% of those requiring Venturi mask (26 out of 29). Using the MMSE in the same subpopulations, Alemanno et al. (2021) found deficits in 55% of those requiring BPAP and in 49% of patients requiring Venturi mask (10 out of 18 and 14 out of 29, respectively). Among studies using multi-domain screenings, one study (Ferrucci et al., 2021) administered in-person the Brief Repeatable Battery of Neuropsychological Test (BRB-NT; Amato et al., 2006); 60% of the sample (*N* = 38) was impaired in at least one subtest. The most frequently impaired cognitive domains were processing speed, visual/verbal short-term memory, long-term memory, and language (especially semantic verbal fluency). Finally, Miskowiak et al. (2021) administered the Screen for Cognitive Impairment in Psychiatry Danish Version (SCIP-D) (Purdon, 2005; Jensen et al., 2015) in the presence of a heterogeneous proportion of patients with deficits, depending on the cut-off considered (62% were globally impaired when considering a less conservative criterion, while 37% when considering a stricter cut-off). The most frequent impairments were in the domains of WM, verbal fluency, and psychomotor speed.

#### Neuropsychological Assessment in Mild Patients

The study investigating *mild* patients assessed cognition through in-person administration of MoCA (Del Brutto et al., 2021). Another study from mixed category assessed mild patients (in-person MoCA and MMSE) reporting NPs outcomes for each disease severity group separately (Alemanno et al., 2021); its findings are therefore reported in this section. MoCA pathological scores were found in 21% (11 out of 52, Del Brutto et al., 2021) and 77% (7 out of 9, Alemanno et al., 2021) of patients. The MMSE scores were pathological in 4 out of 9 patients (44%, Alemanno et al., 2021).

#### Neuropsychological Assessment in Mixed Patients

Finally, studies with *mixed* or unspecified severity samples (*N* = 10) assessed cognition both in-person (*N* = 8) and remotely (*N* = 2). The first-level assessment was performed in 7 out of 10 studies, with MoCA being the most commonly administered test, followed by MMSE and TICS.

The results of two of the studies categorized as mixed (Alemanno et al., 2021; Heyns et al., 2021) are reported here as they reported results separately for disease severity that have been included in the previous sections of this manuscript.

Aiello et al. (2022) administered both MoCA test and the MMSE in-person, dividing patients with COVID-19 in two groups: being those at risk of developing cognitive deficit or not at risk of developing cognitive deficit (RCD + and RCD–). The authors found pathological scores on MoCA in 23% (RCD+, 13 out of 55) and 4% (RCD–, 2 out of 45) of patients. Whereas, MMSE scores were found to be pathological in 20% (RCD+, 11 out of 55) and 2% (RCD–, 1 out of 45) of the sample. Studies administering MoCA test in-person on mixed populations found pathological scores in 80% (62 out of 77, Patel et al., 2021), 30% (30 out of 100, Boesl et al., 2021), and, finally, 17% (22 out of 126, Pilotto et al., 2021) of patients. One study (Mcloughlin et al., 2020) investigated cognition through a modified-version of TICS administered from remote; here, the authors compared the cognitive profiles of COVID-19 patients with and without delirium: mean cognitive scores were similar among the two groups, but exact percentages were not reported by the authors.

Regarding studies with second-level assessment in mixed samples, Almeria et al. (2020) conducted a thorough in-person NPs evaluation. The authors found 12 out of 35 (34%) patients showing cognitive impairments. Specifically, those with mild neurological symptoms (e.g., anosmia or headache) had lower scores on WM tests; patients that needed oxygen therapy had lower scores on verbal and visual memory, attention, WM, processing speed, and EFs. Finally, patients that stayed in the ICU showed lower scores only on EFs. Mazza et al. (2021) administered the Brief Assessment Cognition Schizophrenia (BACS; Keefe et al., 2004) to 130 patients, showing that 16% had pathological scores on at least one function, 17% in two, 14% in three, 11% in four, 5% in five, and 1.5% showing pathological scores in each domain. Finally, Lamontagne et al. (2021) evaluated 50 healthy controls and 50 patients with COVID-19, who were classified into patients with acute COVID-19, Post-Acute Sequelae of COVID-19 (PASC), and post-PASC. After remotely administering the Attention Network Test (ANT; Fan et al., 2002), which evaluates the attentional networks of alerting, orienting, and executive control by means of reaction times, researchers reported a selective impairment only on executive functioning in the PASC phase.

## Discussion

The goal of this review is to provide clinicians with an overview of first- and second-level NPs tests that have been used *de visu* and remotely to assess cognition among patients with COVID-19.

Results from included studies corroborate that cognitive dysfunction is a common feature among patients with SARS-CoV-2. Although the cognitive sequelae of SARS-CoV-2 infection seem consistently captured by both global examinations and domain-specific assessments, vastly different degrees of impairment were found, depending on first- *vs*. second-level tests, modality of administration (i.e., in person *vs.* remote), and disease severity.

The cognitive domains found to be most frequently impaired were EFs, attention, and memory, as assessed both by first- (e.g., Alemanno et al., 2021) and second-level (Ferrucci et al., 2021; Miskowiak et al., 2021) tests.

Regarding first-level tests, studies administering the MoCA found a remarkably higher proportion of pathological scores among moderate patients (Alemanno et al., 2021; Heyns et al., 2021) when compared with severe patients (Alemanno et al., 2021; Heyns et al., 2021). Similarly, studies using the MMSE in severe patients found a relatively low prevalence of pathological scores (Alemanno et al., 2021; Monti et al., 2021), whereas these were much higher in moderate and mild patients (Alemanno et al., 2021). In particular, the prevalence of impairment was consistently lower when assessed through MMSE as compared with MoCA (mild: 4 patients/9 MMSE vs. 7/9 MoCA; moderate: 14/29 MMSE vs. 26/29 MoCA; and severe: 4/31 MMSE vs. 22/31 MoCA; Alemanno et al., 2021). With regards to the lower proportion of cognitive deficits in severe *vs.* moderate patients, it is possible that patients presenting with severe symptomatology (e.g., requiring invasive ventilation), or more aggressive treatments (e.g., intubation) experienced less extensive hypoxic damage to the brain, which is instead typically associated with moderate-to-severe COVID-19 presentations (Alemanno et al., 2021). By contrast, moderate patients might have suffered from hypoxic states for prolonged time, thus showing more severe neurocognitive sequelae (Sasannejad et al., 2019). Furthermore, studies assessing severe patients with COVID-19 may have suffered from a selection bias in that patients with more critical health conditions may have been excluded from the data collection process because the NPs evaluation was not feasible. This may also explain why a lower proportion of cognitive deficits was found among severe patients with COVID-19. Taken together, albeit very preliminary in nature, these findings are in line with previous literature, suggesting that, across patients with COVID-19, MoCA may have higher sensitivity in detecting mild cognitive deficits (Pinto et al., 2019), whereas the MMSE could be more useful for patients who present with severe impairments (Tsoi et al., 2015). With respect to the TICS—administered remotely to either severe (Soldati et al., 2021) or mixed (Mcloughlin et al., 2020) patients, a relatively low prevalence of impaired performance was found, preliminarily suggesting that this test has limited usefulness in this population.

It is worth noting that the proportion of pathological scores within the *mild* category is highly variable among the two studies here included (Alemanno et al., 2021; Del Brutto et al., 2021). The one that reported remarkably high proportions of deficits (Alemanno et al., 2021) has two issues that limit the generalizability of findings: first, the sample size was small (*N* = 9); second, the majority of patients included and assessed were older adults aged 75 years and above (62.56 ± 20.06; mean age and standard deviation [SD]). Therefore, the higher rate of cognitive impairment could be linked to age-related risk factors rather than to the disease itself. This hypothesis seems corroborated by the fact that Del Brutto et al. (2021), who assessed 52 participants aged 59.4 ± 10.6 years, only found 21% of the sample being impaired on MoCA. Taken together, these findings suggest that more sensitive and reliable tests are likely needed to assess cognitive impairments in mild patients.

With regards to the second-level assessment, three studies focus on clinical populations examined with mixed illness severity (Almeria et al., 2020; Lamontagne et al., 2021; Mazza et al., 2021), two studies focused on patients with moderate illness severity (Ferrucci et al., 2021; Miskowiak et al., 2021), and only one was conducted on severely ill patients (Zhou et al., 2020). The included studies mostly evaluated attention and/or EFs using different tests, thus not allowing for direct comparisons. Nonetheless, the following tests were frequently used: Trail Making Test A and B (TMT-A/B; Zhou et al., 2020; Almeria et al., 2020; Miskowiak et al., 2021), Symbol Digit Modality Test (SDMT; Almeria et al., 2020; Ferrucci et al., 2021), Continuous Performance Test (CPT; Zhou et al., 2020), Paced Auditory Serial Addition Task (PASAT; Ferrucci et al., 2021); Digit Forward and Backward, Fluency tests, and Stroop test (Almeria et al., 2020). However, once again, patients with moderate illness severity showed a higher prevalence of cognitive impairment (Ferrucci et al., 2021; Miskowiak et al., 2021) when compared to those with mixed-severity (Almeria et al., 2020; Zhou et al., 2020). Accordingly, Almeria et al. (2020) found that patients requiring O_2_ therapy, but not ICU admission, showed impairment in several cognitive domains (e.g., memory, attention, and EFs) whereas patients who needed to be intubated only showed deficits on EFs.

Drawing definitive conclusions about *mixed* samples is complicated by the fact that patients showed symptoms ranging from mild to severe. Since different illness severities are associated with different cognitive profiles, it remains challenging to disentangle the effect of illness severity on the overall proportion of pathological scores.

Similarly, the lack of studies investigating II-level cognitive deficits in mild populations does not allow us to infer which type of test is more appropriate to characterize the cognitive profile of patients with mild COVID-19 symptoms. Arguably, if such patients present with subtle alterations, domain-specific tests, rather than global screeners, may be more useful in this context.

Some considerations are necessary when discussing the modality of assessment (in-person *vs.* remote). First, studies assessing patients with COVID-19 remotely either used telephone-based tools (Monti et al., 2021; Soldati et al., 2021) or an iPad-based assessment (Zhou et al., 2020). In one of these studies where MMSE was administered remotely to patients with severe COVID-19 (Monti et al., 2021), the proportion of patients found to be impaired was lower when compared with a study where the same test was administered in-person to patients with severe COVID-19 (Alemanno et al., 2021). This raises the possibility that remote NPs assessment may underestimate the actual prevalence of cognitive deficits among patients with COVID-19, especially when a global screener is used.

The studies hereby reviewed present several methodological limitations, the main one being the inconsistency of disease severity classifications across studies. A clearer consensus categorization is needed to be able to compare results across studies. Additionally, several studies did not include relevant demographic characteristics of patients enrolled (e.g., years of education, medical comorbidities, or disease duration). This hampers a proper interpretation of results and makes comparison between study populations fraught with problems. Finally, most studies were significantly underpowered, including less than 30 participants (*N* = 4).

## Conclusion

Our review of the literature highlights the following points: (i) The MoCA may be able to catch subtle cognitive alterations, at least on patients with moderate COVID-19, whereas the MMSE is more indicated for severe cognitive deficits; (ii) although several second-level NPs assessments have consistently indicated the presence of attentive and executive deficits, the limited amount of available evidence does not allow to draw specific conclusions, and research is needed to deeply characterize cognitive deficits following COVID-19 infection; and (iii) in-person NPs evaluation seems to be the best choice to investigate cognitive deficits in this population.

Despite the low methodological rigor of this nascent field of research, the early identification and characterization of cognitive consequences following COVID-19, across all degrees of disease severity, remains of paramount importance. While the older population is certainly that with the greatest vulnerability to cognitive decline, the possible downstream cognitive consequences of COVID-19 infection in younger, mild, or asymptomatic cases are emerging (Ortelli et al., 2022). Based on our review, we recommend the implementation of both baseline and follow-up NPs screenings that are consistent with disease severity classification.

Finally, because cognition actively impacts an individual’s capacity to work effectively, drive, manage finances, participate in daily family activities or make informed decisions, specific prevention and intervention programs that remediate cognitive deficits will be an important next step to achieve independent functioning and improved quality of life among many patients who endured COVID-19.

## Author Contributions

AD, BB, AN, IL, and PB: conceptualization. AD, BB, AN, IL, LN, and GF: investigation. BB, AN, IL, and LN: methodology. AD, BB, AN, and IL: writing – original draft. AD, AN, IL, LN, and GF: formal analysis. AD, BB, AN, IL, EZ, NS, and PB: writing – review and editing. All authors contributed to the article and approved the submitted version.

## Conflict of Interest

The authors declare that the research was conducted in the absence of any commercial or financial relationships that could be construed as a potential conflict of interest.

## Publisher’s Note

All claims expressed in this article are solely those of the authors and do not necessarily represent those of their affiliated organizations, or those of the publisher, the editors and the reviewers. Any product that may be evaluated in this article, or claim that may be made by its manufacturer, is not guaranteed or endorsed by the publisher.

## Footnotes

1
https://rayyan.qcri.org/welcome
