# Supplementary material for: Cognitive Assessment in SARS-CoV-2 Patients: A Systematic Review
Source: Front Aging Neurosci. 2022 Jul 1;14:909661. doi: 10.3389/fnagi.2022.909661 (PMC9283975; doi:10.3389/fnagi.2022.909661)
Supplement: Supplementary file 1 [file Table_1.DOCX]

Supplementary Table 1. Categorization of included studies based on cognitive test or domain.

| **First**  **Author** | **Time of Assessment** | **Total Patients’ Size** | **Disease Severity** | **% Patients with Deficits** |
| --- | --- | --- | --- | --- |
|  |  |  |  | **(or Mean & SD)** |
| ***MOCA*** |  |  |  |  |
| Aiello et al. | Mean 74, SD 41 days post discharge | 100 | 55 at risk, 45 not at risk | at risk: 24%, not at risk: 4% |
| Alemanno et al. | 5-22 days post symptom onset | 87 | 31 severe, 18 bpap, 29 venturi, 9 mild | 72%, 94%, 90%, 78% |
|  |  |  |  |  |
| Boesl et al. | Mean 185 days | 100 | mixed | 30% |
| Del Brutto et al. | 6 months after symptoms onset | 52 | mild | 21% |
| Heyns et al. | N/A | 38 | 15 severe, 23 moderate | 47%, 61% |
| Latronico et al. | 3 , 6, 12 months post discharge | 98 | severe | 25% |
| Patel et al. | 72 hours after admission in rehab clinic | 77 | mixed | 81% |
| Pilotto et al. | 6 months post discharge | 126 | mixed | 18% |
| Solaro et al. | N/A | 32 | moderate | 37% |
|  |  |  |  |  |
| ***MMSE*** |  |  |  |  |
| Aiello et al. | Mean 74, SD 41 days post discharge | 100 | 55 at risk, 45 not at risk | at risk: 20%, not at risk: 2% |
| Alemanno et al. | 5-22 days post symptom onset | 87 | 31 severe, 18 bpap, 29 venturi, 9 mild | 13%, 56%, 48%, 44% |
| Monti et al. | 51-61 days post discharge |  |  | 3% |
| Pistarini et al. | 10 days post symptom onset | 27 | 20 PCR+, 7 PCR- | 35%, 5% |
|  |  |  |  |  |
| ***Executive Functioning*** | |  |  |  |
| Almeria et al. | 10-35 days post discharge | 35 | mixed | Digit Forward: N/A |
|  |  |  |  | Digit Backward: 9% |
|  |  |  |  | Stroop: 3% |
|  |  |  |  | Phonetic Fluency: 11% |
|  |  |  |  | Semantic Fluency: 6% |
| Ferrucci et al. | Mean 133 / 37 days post discharge | 38 | moderate | Word List Generation: 8% |
| Mazza et al. | one month after hospital discharge | 130 | mixed | Verbal Fluency: 32 % |
|  |  |  |  | Working Memory: 24 % |
|  |  |  |  | Executive Functions: 50% |
| Miskowiak et al. | 3 monts post discharge | 29 | moderate | Working Memory: 18.2 (4.2); |
|  |  |  |  | Verbal fluency test: 14.3 (4.7); |
| Zhou et al. | N/A | 29 | N/A | DST: 0% |
|  |  |  |  |  |
| ***Attention*** |  |  |  |  |
| Almeria et al. | 10-35 days post discharge | 35 | mixed | TMT-A: 3% |
|  |  |  |  | TMT-B: 9% |
|  |  |  |  | Symbol Digit Span: 3% |
| Ferrucci et al. | Mean 133 / 37 days post discharge | 38 | moderate | Symbol Digit Span: 42% |
|  |  |  |  | PASAT: 11% |
| Mazza et al. | one month after hospital discharge | 130 | mixed | Attention and Information Processing: 33% |
| Miskowiak et al. | 3 monts post discharge | 29 | moderate | TMT-B: 116.2 (65.0) |
| Zhou et al. | N/A | 29 | N/A | TMT-A: 0% |
|  |  |  |  | SCT: 0% |
|  |  |  |  | CPT: lower correct number and higher missing number in CPT2 e CPT3 compared to HC |
